# Does the expensive brain hypothesis apply to amphibians and reptiles?

**DOI:** 10.1186/s12862-023-02188-w

**Published:** 2023-12-19

**Authors:** Zitan Song, Michael Griesser, Caroline Schuppli, Carel P. van Schaik

**Affiliations:** 1 Comparative Socioecology group, Department for the Ecology of Animal Societies, Max Planck Institute for Animal Behavior, 78467 Konstanz, Germany; 2 Department of Biology, University of Konstanz, 78467 Konstanz, Germany; 3 Centre for the Advanced Study of Collective Behaviour, University of Konstanz, 78467 Konstanz, Germany; 4 Department of Collective Behavior, Max Planck Institute of Animal Behavior, 78467 Konstanz, Germany; 5 Development and Evolution of Cognition Group, Max Planck Institute for Animal Behavior, 78467 Konstanz, Germany; 6 Department of Evolutionary Biology and Environmental Studies, University of Zurich, Zurich, 8057 Switzerland; 7 Center for the Interdisciplinary Study of language Evolution, University of Zurich, Zurich, 8057 Switzerland

**Keywords:** Ambient temperature, Brain size, Ectothermy, Expensive brain, Seasonality, Nocturnality, Hibernation, Brumation

## Abstract

Vertebrate brains show extensive variation in relative size. The expensive brain hypothesis argues that one important source of this variation is linked to a species’ ability to generate the energy required to sustain the brain, especially during periods of unavoidable food scarcity. Here we ask whether this hypothesis, tested so far in endothermic vertebrates, also applies to ectotherms, where ambient temperature is an additional major aspect of energy balance. Phylogenetic comparative analyses of reptiles and amphibians support the hypothesis. First, relative brain size increases with higher body temperature in those species active during the day that can gain free energy by basking. Second, relative brain size is smaller among nocturnal species, which generally face less favorable energy budgets, especially when maintaining high body temperature. However, we do not find an effect of seasonal variation in ambient temperature or food on brain size, unlike in endotherms. We conclude that the factors affecting energy balance in ectotherms and endotherms are overlapping but not identical. We therefore discuss the idea that when body temperatures are seasonally very low, cognitive benefits may be thwarted and selection on larger brain size may be rare. Indeed, mammalian hibernators may show similarities to ectotherms.

**Supplementary Information:**

The online version contains supplementary material available at 10.1186/s12862-023-02188-w.

## Introduction

Brains have unusually high energy costs per unit tissue [1, 2], linked to the high energetic demands of synaptic transmission [3]. Because larger brains provide numerous adaptive sensorimotor and cognitive benefits in a wide range of conditions, the expensive brain hypothesis [4, 5] proposes that these high costs constrain relative brain size, despite potential fitness advantages due to the cognitive benefits of having larger brains. Specifically, it predicts that smaller brains may reflect the combined effect of periods of unavoidable reductions in net energy intake [4, 5] and competition between energy allocation to the brain and other expensive functions [6], most prominently growth and reproduction [7]. These predictions received broad empirical support among endothermic taxa. First, brain size shows well-documented tradeoffs with growth and reproduction in many lineages [5, 8]. Second, brain size is reduced where endothermic animals experience reduced energy intake during seasonal scarcity [9] or periods of unavoidable starvation [10], as on small islands. Third, the high costs of brains also suggest that offspring of larger-brained species are incapable of fulfilling the brain’s energy demands on their own, and thus need parental provisioning to support the growth and differentiation of their brains [11]. Hence, the ability to provision the young may have become a constraint on brain size evolution. This developmental version of the hypothesis is supported by strong correlated evolution between the amount of parental provisioning and brain size among birds [12]. It also explains the finding that larger-brained species have larger neonates in mammals [5].

The above studies were predominantly conducted on endothermic species. The tradeoffs between brain size and growth rate (and thus age at maturity) and reproductive output reported for endotherms are also apparent in ectotherms (anurans: [13, 14]; fishes: [15]; reptiles: [16]). Likewise, as in mammals [5] and birds [12, 13], larger-brained species tend to produce larger offsprings in lineages without post-hatching provisioning (frogs: [17]; lizards [Song et al., unpublished]; chondrichthyan fishes: [18]). However, virtually no studies have assessed whether the predicted negative effects of periodic or episodic food scarcity on brain size are also found in ectotherms.

The aim of this paper is to contribute to filling this gap. Effects of periodic scarcity are expected given that ectotherms, unlike endotherms, usually are incapable of sustained activity [19], which should make them more vulnerable to fluctuations in net food intake and the resulting periods of negative energy balance, which select for smaller brain size. The only existing study [20] found that seasonality in food was linked to reduced brain size in frogs, but their seasonality index could not distinguish between the effects of low food and of low ambient temperature. Here we develop predictions for comparative tests with ectothermic tetrapods (amphibians and reptiles), given that endothermy evolved on land (e.g. [21]) and land-based vertebrates can become at least partly independent from ambient temperatures relative to fully aquatic ectotherms (fishes) by basking, changing posture or moving between temporarily favorable microhabitats. We will test three predictions that are unique to ectotherms.

Endotherms can generate heat internally and use this to maintain high and especially stable body temperature (homeothermy). In contrast, ectotherms primarily rely on external sources of heat to regulate their body temperature, directly or through basking (especially found in reptiles: [22], and occasionally in anurans: [23]), which does not require metabolically generated energy. As a result, their energy balance is not solely dependent on food availability and intake but is also influenced by body temperature. The first two predictions therefore relate to the effects of body temperature on brain size (cf. [24, 25]).

The physiological processes of endotherms run at a higher and more stable body temperature than in the great majority of ectotherms [26], with only lizards showing partial overlap with mammals [27]. A higher body temperature has the advantage that biochemical processes run reliably fast. All physiological processes follow the Q_10_ rule, which means that for every 10° C rise in temperature biochemical processes run ca. 2–3 times faster [28]. Many ectotherms have evolved adaptations to remain active at far lower body temperatures than endotherms can (e.g., [23]). However, remaining active at such low body temperatures must in itself be physiologically costly. The Q_10_ rule indicates that we should expect that major reductions in temperature slow down nerve conduction speed and thus may have detrimental effects on higher processing in the brain of ectotherms. Thus, avoiding these negative effects requires various energetically costly structural (e.g. adding mitochondria) or biochemical adaptations (e.g. having a variety of enzymes allowing performance at a broad range of temperatures) [26]; cf. [29, 30]. This explains why different species are able to maximize physiological functions at highly different temperatures [28]. Ectotherms with high body temperature (T_b_) during activity therefore can approach the optimum temperature range, but the lower mean T_b_ gets, the higher the physiological price of remaining active. Thus, ectotherms that are active at lower T_b_ should be energetically more constrained than those active at higher T_b_, and this should leave less energy to be allocated to the brain. We therefore predict that higher T_b_ will be accompanied by increased relative brain size (**Prediction 1**).

Basking allows species to have T_b_ well above ambient temperatures during their active period. However, strictly nocturnal species cannot bask, and therefore can only maintain higher T_b_ relative to ambient temperatures by increasing activity levels. However, doing this requires energy and would therefore negatively affect the energy balance. As a result, we expect smaller relative brain size in nocturnal species (**Prediction 2**).

Unlike endotherms, ectotherms may generally be unable to generate the high and stable energy food intake needed to sustain larger brains during times of food scarcity. We expect this effect to be even stronger in ectotherms due to their reduced activity levels and mobility. Thus, we predict that seasonal food scarcity will be linked to smaller brain sizes (**Prediction 3**). This seemingly obvious prediction is somewhat speculative, however. Among endotherms, energy requirements remain approximately constant during periods of scarcity, or even increase when food scarcity is accompanied by lower ambient temperatures (T_a_) (e.g. [31, 32]). In ectotherms, in contrast, energy needs may be reduced during periods of scarcity. They tend to becomes less active (or even enter brumation) and have reduced metabolic rates during periods of low temperature (cf. [33–35]) or even merely during food scarcity [36]. Thus, food intake and energy requirements may vary approximately in parallel and no energy scarcity for the brain ensues, and no brain size reduction might evolve whenever seasonal food scarcity is accompanied by colder temperatures.

We tested these three predictions in a comparative study that leveraged published data on amphibians and reptiles.

## Methods

### Materials and methods

#### Morphological data

We conducted an extensive literature search to gather the data for this study. We used the ISI Web of Science to search for all articles published before November 2022 using the search terms ‘reptile’, ‘rhynchocephalia’, ‘lizard’, ‘snake’, ‘squamata’, ‘amphibia’, ‘anura’, ‘frog’, ‘caudata’, ‘salamander’ and ‘gymnophiona’, combined with ‘brain size’, ‘brain mass’, ‘brain weight’, ‘encephalization’ and ‘brain-to-body ratio’. We excluded the terms ‘dinosaur’, ‘fossil’ and ‘extinct’. In total, we found 53 studies for reptiles and 63 studies for amphibians, which we checked for completeness using Google Scholar using the same search terms, and checking for studies that cited studies we already had. We took the mean values of brain mass (g) and body mass (g) from the same specimens (of either sex) of all species, if needed by averaging values from different studies, while excluding duplicate reports. In total, we collected brain mass and body mass of 160 lizard species, 28 snake species, the only extant rhynchocephalian, one worm lizard, 123 frog species and 54 salamander species. We used several major sources [16, 17, 37], but also always checked the original sources. The full data set is available in the Supplementary Materials.

#### Activity patterns

We collected data on activity period of the species with brain size data, and distinguished between a lack of basking opportunities (which pits nocturnal species against both diurnal and cathemeral ones, which were therefore combined into one category). For 358 of the 367 species, we were able to categorize their activity periods. We used several major sources [38–40], but also always checked the original sources. The full data set is available in the Supplementary Materials.

#### Ecological data: ambient temperature and food availability

Data of the geographic ranges for reptiles were obtained from recently published data source [41] and for amphibians from the IUCN [42]. We use the mean annual temperature (BIO1), as defined by [43] (data downloaded from WorldClim website: www.worldclim.org/data/bioclim) to characterize mean ambient temperature (T_a_), and BIO4 as the measure of temperature seasonality. We calculated the temperature measures for the whole distribution range for each species by taking the average of all 5 × 5 km cells in their range.

To characterize seasonality in food abundance, we relied on vegetation measures linked to plant growth, because the abundance of both young foliage and insects, the main food of replies and amphibians, peaks at periods of high plant productivity [41]. Thus, we used the coefficient of variation in monthly average values of the NDVI index (normalized difference vegetation index), which estimates the abundance of chlorophyll based on Moderate Resolution Imaging Spectroradiometer (MODIS) data (MOD13C2v006). These measures were calculated from the entire geographic range (using 0.05 deg CMG grid cells, which are 5.6 × 5.6 km) for 20 years from 2001 to 2020. Because detailed geographic ranges were missing for a few species, we had mean temperature and NDVI data for 363 and 361 species, respectively.

#### Body temperature

Records of T_b_ were collected from published sources (as detailed in the Data accessibility section). We only included mean T_b_ records during the animals’ active period, and therefore excluded both data from animals that were brumating and records of preferred T_b_ in lab studies. For species with multiple reports, we took the average of the mean T_b_ for each population. In total, we collected mean body temperature for 48 amphibian and 105 reptile species. The major source is [40], but we also always checked the original sources. The full data set is available in the Supplementary Materials. We found too few reliable data on temporal variation in T_b_ of active animals to be able to include this measure in our analyses.

#### Statistical analyses

We implemented all statistical analyses in R 4.1.1 [44], using the package phylolm [45] to control for any effects of phylogenetic non-independence. The figures were generated in the package ggplot2 [46]. To visualize the results, the figures show residual brain size, even though the analyses were based on absolute brain size while controlling for body mass as a covariate.

To test the predicted correlated evolution between relative brain size and T_b_ (P1) and nocturnality (P2), we fitted a phylogenetic generalized least squares regression (PGLS) with absolute brain mass (log 10 transferred) as the dependent variable and body mass (log 10 transferred), taxon (reptile or amphibian), activity period (diurnal and cathemeral species have opportunities for basking or use the higher ambient temperatures accompanying daytime, and were therefore combined, whereas nocturnal ones do not), mean T_b_, as well as the interaction between activity period and mean T_b_. We first tested the overall fit of the model by comparing the full model, including the predictors (activity period and T_b_), and control variables (body mass and taxon) with the null model (which only includes the control variables) using a likelihood ratio test [47]. Subsequently, we extended the likelihood ratio test (LRT) by comparing the full model with a model that also included an interaction between activity period and mean T_b_. No multicollinearity was observed among the four independent variables (Table S1). Visual inspection of all model fits confirmed that they satisfied model assumptions (including normally distributed model residuals, and homogeneity of the variance [48]). Additionally, where the interaction between body temperature and activity period showed a significant effect on brain, we separated the PGLS model by activity period in order to fully investigate the predictions 1 & 2, i.e., whether T_b_ had a positive or negative effect on brain size depending on activity period. To control for phylogenetic uncertainty in tree reconstruction, we used a recently published time-calibrated multi-tree phylogeny for reptiles [49] and amphibians [50], with randomly selected 100 trees to run PGLS models for each lineage.

To test the predicted correlated evolution between relative brain size and seasonality in food (P3), we fitted PGLS models with absolute brain mass (log 10 transferred) as the dependent variable and body mass (log 10 transferred), taxon (reptile or amphibian), as well as the activity period, mean and seasonality in ambient temperature, and the mean and CV (coefficient of variation) of NDVI per year as independent variables (the latter all untransformed). Because of multicollinearity among the climate and NDVI variables (see Table S1), we did several independent analyses with one of the collinear variables removed. As above, we visually assessed whether model assumptions were satisfied. This same analysis was repeated for a subset of species for which we had mean body temperatures, which was also included as an independent variable (see Table S3).

## Results

**Fig. 1:**
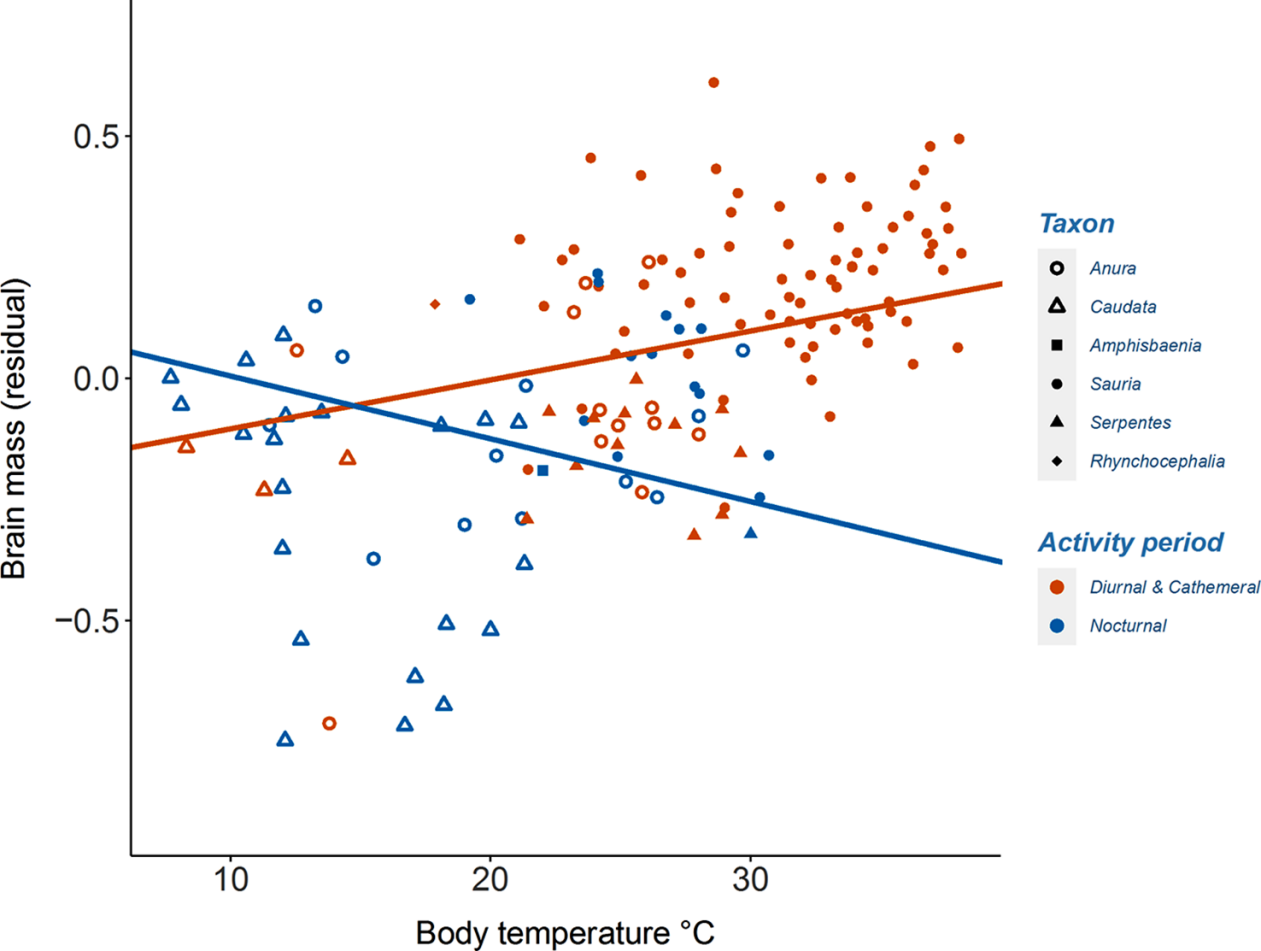
Relative brain size increases with mean body temperature in diurnal & cathemeral species, while decreases in nocturnal species. Lines were depict the PGLS equations

We first examined the effect of T_b_ (mean body temperature) on brain mass. Including activity period and T_b_ into the model significantly improved model fit, as evidenced by a significant LRT (X^2^ = 6.649, p = 0.036). Furthermore, the interaction between activity period and T_b_ also yielded a significant enhancement to the model compared to the one without this interaction term (LRT, X^2^ = 11.626, p < 0.001). As documented in Table 1, there was a significant effect of activity period, but its effect interacted with that of T_b_: in diurnal or cathemeral species, which have opportunities to warm their bodies through basking, relative brain size increased with T_b_, whereas in nocturnal species it decreased (Fig. 1). Separate analyses for these two categories of activity periods showed that both effects were significant (Table S2). For diurnal and cathemeral species, this result confirms the correlated evolution between relative brain size and T_b_ (Prediction 1). In contrast, nocturnal species had smaller relative brain sizes, but the interaction effect revealed that this only held for species with higher T_b_ (Fig. 1), which confirms the predicted negative correlation between nocturnality and relative brain size (Prediction 2).

**Table 1:** PGLS analyses of the effects of body temperature and activity period on brain size, while controlling for various confounding effects. Variables with significant effects (P < 0.05) are highlighted in in bold. The 95% confidence intervals for the 100 trees are shown in brackets. λ is Pagel’s lambda

	Estimate	se	t	P
**All species (n = 153)**				
Intercept	0.767 (0.765, 0.769)	0.175 (0.173, 0.177)	4.401 (4.348, 4.453)	< 0.001 (<0.001, <0.001)
**Body mass (log-10)**	0.552 (0.552, 0.552)	0.020 (0.020, 0.020)	27.843 (27.806, 27.881)	< 0.001 (<0.001, <0.001)
Taxon *(Reptile)*	0.272 (0.271, 0.274)	0.234 (0.230, 0.239)	1.168 (1.150, 1.185)	0.247 (0.240, 0.254)
**Activity period** **(nocturnal)**	0.343 (0.340, 0.346)	0.138 (0.137, 0.138)	2.492 (2.476, 2.508)	0.014 (0.013, 0.015)
Body temperature (T_b_)	0.007 (0.007, 0.008)	0.004 (0.004, 0.004)	1.949 (1.927, 1.972)	0.055 (0.052, 0.058)
**AP (nocturnal) x T**_**b**_	-0.019 (-0.019, -0.019)	0.006 (0.006, 0.006)	-3.430 (-3.446, -3.414)	<0.001 (<0.001, <0.001)
			λ = 0.796 (0.785, 0.807); R^2^ = 0.846 (0.845, 0.846)	

We found no correlated evolution between relative brain size and seasonality in food (disconfirming Prediction 3). While controlling for the effects of body size, taxon (reptiles versus amphibians), and activity period, no effect was found for either mean ambient temperature or the mean or CV in the NDVI (Table 2) nor of seasonality of ambient temperature (Table S3) in the full sample (n = 350 species). When we repeated this analysis for the subset of species (n = 148) for which we had information on mean T_b_, the results remained the same (Tables S4 & S5).

**Table 2:** PGLS analyses of the effects of the ambient environment including average ambient temperature, average and CV of NDVI on brain size, while controlling for various confounding effects. Variables with significant effects (P < 0.05) are highlighted in in bold. The 95% confidence intervals for the 100 trees are shown in brackets. λ is Pagel’s lambda

	Estimate	se	t	P
**CV NDVI (n = 350)**				
Intercept	0.960 (0.959, 0.961)	0.171 (0.170, 0.173)	5.618 (5.573, 5.663)	< 0.001 (< 0.001, < 0.001)
**Body mass (log-10)**	0.536 (0.535, 0.536)	0.016 (0.016, 0.016)	33.284 (33.259, 33.308)	< 0.001 (< 0.001, < 0.001)
Taxon (R*eptile*)	0.327 (0.326, 0.328)	0.240 (0.238, 0.241)	1.365 (1.354, 1.375)	0.174 (0.171, 0.177)
**Activity period** **(nocturnal)**	-0.055 (-0.056, -0.055)	0.026 (0.026, 0.026)	-2.124 (-2.140, -2.107)	0.035 (0.034, 0.037)
Average ambient temperature	-0.002 (-0.002, -0.002)	0.003 (0.003, 0.003)	-0.915 (-0.928, -0.901)	0.362 (0.355, 0.369)
Average NDVI per year	0.067 (0.066, 0.067)	0.069 (0.069, 0.070)	0.962 (0.952, 0.972)	0.338 (0.332, 0.343)
CV NDVI per year	-0.002 (-0.002, -0.002)	0.001 (0.001, 0.001)	-1.057 (-1.068, -1.046)	0.292 (0.287, 0.297)
			λ = 0.757 (0.753, 0.761); R^2^ = 0.770 (0.770, 0.770)	

As elaborated in the supplementary analyses, these results were robust against examining the role of possible confounding variables (other aspects of the ecological niche or major differences in body plan) and using alternative ways of calculating existing variables (using the centroid rather than the mean of the geographic range).

## Discussion

In this study, we tested three predictions of the expensive brain hypothesis on relative brain size in tetrapod ectotherms. Overall, we found good support for the effect of body temperature (P1) and activity period (P2), but found no evidence for the hypothesized effect of seasonality in food (P3).

In species potentially able to bask (i.e., diurnal and cathemeral ones), mean body temperature while active (T_b_) was positively correlated with brain size. This pattern supports the idea that species able to achieve higher T_b_ from environmental sources have, other things being equal, a more favorable energy balance (P1). In nocturnal species, brain size decreased with T_b_ (P2), supporting the idea that costly biochemical adaptations or muscle activity [51] are needed to maintain a higher T_b_ during the night, when ambient temperatures are reduced and no basking is possible. Doing so must negatively affect energy balance, and thus brain size. In general, reptiles in our dataset live in warm habitats (Figure S1; see also [41]) and accordingly their T_b_ during their active period are above the mean ambient temperature (Figure S2). In both reptiles and amphibians, T_b_ is also higher in diurnal and cathemeral species than in nocturnal species (Figure S2), reflecting both basking opportunities and warmer ambient temperatures. Nocturnal reptiles live in warmer habitats than diurnal or cathemeral ones but this difference is not found in amphibians (Figure S1). This pattern reflects that reptiles are both more active and more likely to bask when diurnal or cathemeral, and consequently achieve favorable T_b_ even in colder habitats [52, 53], whereas amphibians are capable of remaining active at far lower body and ambient temperatures [23].

Overall, these findings therefore show that quadrupedal ectotherms often have smaller brains when they have higher energy costs. For nocturnal reptiles these costs may be higher because they cannot bask and remain active during periods of lower T_b_, whereas for amphibians they are higher when they live in cooler habitats (and cannot bask: many amphibians rapidly dehydrate when they bask: [23]). This later finding echoes suggestive findings in fishes that noted a correlation between seawater temperatures and relative brain size, although food scarcity may be even more responsible ([54], see also [24]).

We found no effect of seasonality in food availability on brain size (P3), unlike in endotherms. Of course, the measure of seasonality in food abundance was crude (landscape-level NDVI) and we also lacked estimates of actual food intake, unlike in primate studies (e.g., [55]). Thus, the rejection of the prediction may be a false negative. Alternatively, it is likely that the periods of low food intake are accompanied by low temperatures and thus by low activity levels and metabolism, since seasonal variation in ambient temperature and in NDVI were highly correlated. However, the same thing may also happen in tropical habitats when food is scarce [33, 36]. Either way, net energy intake and energy requirements may therefore fluctuate roughly in parallel, which would explain the lack of a negative impact of seasonality in food abundance.

These results therefore suggest it is too early to conclude that seasonal food scarcity affects brain size in ectotherms in the same way as in endotherms. The study that found this, in a sample of subtropical frogs ([20]), did not control for seasonality in ambient temperatures, which might explain their result. Most endotherms remain active and are more likely to respond cognitively by shifts in diet or foraging strategy, even if their brain size is somewhat reduced. However, those endotherms that respond to food scarcity physiologically by hibernating have far smaller brains than non-hibernators [56]. This strategy, called brumation in ectotherms, is widespread among ectotherms, and may thus explain the reduced brain size of brumating species, as found in Anura [57]. However, in constrast to the main endotherm pattern, we found that ectotherms that remain active at lower T_b_ have smaller brains, no doubt linked to reduced metabolism. This makes them more tolerant of longer periods of starvation compared to endotherms [58], just like hibernating mammals tend to have better survival than non-hibernating ones [59]. Thus, whether it is due to food scarcity or colder temperatures, many ectotherms appear to respond to unfavorable periods by reducing metabolism, unlike in endotherms [e.g., [31, 32]].

This non-cognitive strategy of coping with food scarcity is entirely consistent with the expensive brain hypothesis. However, the expensive brain hypothesis assumes that brains are as large as the species can afford energetically, and therefore that larger brains bring fitness benefits in terms of perception, cognition, and action (review: [11]). In contrast, if these processes are much slowed down by lower T_b_, such fitness benefits may not accrue. For instance, even a modest reduction in incubation temperature (from 22^0^ to 16^0^C) has been shown to affect subsequent learning in a scincid lizard [60], echoing earlier results on learning in fishes acclimated at lower water (and thus body) temperatures [61, 62]. In that case, larger brains would not bring cognitive benefits, and selection could only rarely favor brain size increases beyond a size that supports those functions that show the steepest marginal effects on fitness and remain possible at lower T_b_.

To test this alternative explanation for the reduced brain size in species with greater seasonal variation in ambient temperature we would need more independent evidence of T_b_ effects on perception and cognition, in particular learning and memory, of animals active at low T_b_. However, the fit with predictions from the expensive brain hypothesis for the basking taxa, which have higher T_b_, especially in the more active reptiles (cf. Figure S2), suggests this alternative may especially apply to taxa with the lowest T_b_ and living in the coolest habitats, and thus in particular, though not exclusively, to amphibians.

This alternative explanation may also hold for hibernating endotherms. In them, the major programmed and long-term reduction in T_b_ and all metabolic processes are correlated with strongly reduced relative brain size [56]. The expensive brain hypothesis suggests this is due to less favorable energy balance during the long period of hibernation, when the organism lives off its accumulated body fat. The alternative interpretation is that brains lose learned skills during long cold periods, thus preventing selection in favor of larger brains. Although the empirical evidence is curiously mixed, various results indicate that the price for having this physiological adaptation against food scarcity may be that the long period of hypothermia due to hibernation reduces cognitive performance. Thus, various studies report clear negative effects of prolonged hypothermia on memory [63–66], presumably linked to the documented pronounced seasonal reductions in brain activity [67], neural connectivity [68], or even overall brain size [69]. Moreover, emergence from hibernation is accompanied by pronounced synaptic remodeling in the hypothalamus, suggesting high relearning activity [68]. Indeed, ground squirrels show far better learning performance right after emerging from hibernation than a month later [70] or than control individuals prevented from hibernating [71]. This pattern is expected if hibernation affects the retention of learned knowledge and motor skills. Such a negative effect of long-term cooling may apply even more to ectotherms, given that brumation, the passive reduction in body temperature leading to immobility, differs from mammalian hibernation by not being occasionally interrupted by arousal [72].

Although these results are partly contradictory, they suggest that in organisms forced to remain active at unusually low T_b_, such as amphibians tied to water and unable to bask, like all Caudata and many Anura [23], the benefits of larger brain size do not accrue as much as to reptiles, with their higher T_b_ and opportunities for basking. As a result, selection would more rarely favor larger brains in organisms forced to live at low T_b_, unless they live in unusually food-rich environments. We hope that future work will help to examine this alternative hypothesis.

Clearly, the comparative tests reported here cannot be the final word. First, limits on the availability of brain data forced us to exclude turtles and crocodilians among reptiles and caecilians among amphibians. Second, mean T_b_s were available only for a fraction of the species. Third, in the absence of data on seasonality in food or climate of the actual habitats inhabited by particular species, we relied on regional measures of seasonality, in particular variation in NDVI (which reflects variation in plant production, and thus insect availability). Nonetheless, we feel the results were robust enough to justify more detailed follow-up studies.

### Electronic supplementary material

Below is the link to the electronic supplementary material.

**Supplementary Material 1:** Supplementary materials

**Supplementary Material 2:** Dataset

**Supplementary Material 3:** R code

## Data Availability

All data are available in the supplementary material.
