# Supplementary material for: Does the expensive brain hypothesis apply to amphibians and reptiles?
Source: BMC Ecol Evol. 2023 Dec 19;23:77. doi: 10.1186/s12862-023-02188-w (PMC10729550; doi:10.1186/s12862-023-02188-w)
Supplement: Supplementary file 1 — Supplementary Material 1: Supplementary materials [file 12862_2023_2188_MOESM1_ESM.docx]

**Does t****he expensive brain hypothesis apply to amphibians and reptiles?**

Zitan Song^1^, Michael Griesser^2, 3^^, 4^, Caroline Schuppli^5^ & Carel P. van Schaik^1, 6, 7*^

**Supplementary Materials**

**Tables**

Table S1. Variance inflation factor (VIF) analyses of all parameters included in the model test prediction 1,2&3.

| **Predictors** | **Model for P1 &2** | **Model for P3** | **Model for P3 with temperature seasonality** |
| --- | --- | --- | --- |
| Body mass (log-10) | 1.011 | 1.023 | 1.022 |
| Taxa | 1.033 | 1.009 | 1.010 |
| Activity period | 1.015 | 1.023 | 1.030 |
| Body temperature | 1.020 |  |  |
| Average temperature |  | 1.622 | 1.928 |
| Temperature seasonality (BIO4) |  |  | 3.324 |
| Average NDVI |  | 1.253 | 1.738 |
| CV NDVI |  | 1.758 | 2.195 |

Table S2. PGLS analyses of the effects of mean T_b_ on brain size for diurnal & cathemeral (basking) and nocturnal (non-basking) species, respectively, while controlling for various confounding effects. Variables with significant effects (P<0.05) are highlighted in in bold. The 95% confidence intervals for the 100 trees are shown in brackets.

|  | **Estimate** | **se** | **t** | **P** |
| --- | --- | --- | --- | --- |
| ***Basking species (n = 104)*** | | | | |
| *Intercept* | 0.820 (0.819, 0.821) | 0.139 (0.137, 0.140) | 5.923 (5.872, 5.974) | <0.001 (<0.001, <0.001) |
| **Body mass** **(log-10)** | 0.519 (0.518, 0.519) | 0.021 (0.021, 0.021) | 24.460 (24.427, 24.493) | <0.001 (<0.001, <0.001) |
| Taxon (*Reptile*) | 0.225 (0.224, 0.226) | 0.178 (0.176, 0.180) | 1.268 (1.256, 1.280) | 0.208 (0.204, 0.213) |
| **Body temperature** | 0.009 (0.009, 0.009) | 0.004 (0.004, 0.004) | 2.300 (2.284, 2.316) | 0.024 (0.023, 0.025) |
| λ = 0.642 (0.633, 0.651); R^2^ = 0.862 (0.861, 0.862) | | | | |
| ***Non-basking species (n = 49)*** | | | | |
| *Intercept* | 1.089 (1.088, 1.091) | 0.178 (0.175, 0.181) | 6.162 (6.061, 6.264) | <0.001 (<0.001, <0.001) |
| **Body mass (log-10)** | 0.606 (0.606, 0.607) | 0.043 (0.043, 0.043) | 13.965 (13.944, 13.987) | <0.001 (<0.001, <0.001) |
| Taxon (*Reptile*) | 0.221 (0.216, 0.225) | 0.224 (0.217, 0.231) | 1.021 (0.976, 1.066) | 0.325 (0.302, 0.348) |
| **Body temperature** | -0.014 (-0.014, -0.014) | 0.007 (0.007, 0.007) | -2.155 (-2.164, -2.146) | 0.037 (0.036, 0.037) |
| λ = 0.611 (0.585, 0.637); R^2^ = 0.813 (0.813, 0.814) | | | | |

Table S3. PGLS analyses of the effects of the ambient environment (including average and seasonality in ambient temperature) on brain size, while controlling for various confounding effects. Variables with significant effects (P<0.05) are highlighted in in bold. The 95% confidence intervals for the 100 trees are shown in brackets.

|  | **Estimate** | **se** | **t** | **P** |
| --- | --- | --- | --- | --- |
| ***Ambient temperature n = 355*** | | | | |
| Intercept | 1.001 (1.000, 1.002) | 0.169 (0.167, 0.170) | 5.942 (5.892, 5.992) | <0.001 (<0.001, <0.001) |
| **Body mass (log-10)** | 0.537 (0.537, 0.537) | 0.016 (0.016, 0.016) | 33.628 (33.603, 33.653) | <0.001 (<0.001, <0.001) |
| Taxon (R*eptile*) | 0.318 (0.317, 0.318) | 0.239 (0.238, 0.241) | 1.328 (1.318, 1.338) | 0.186 (0.182, 0.189) |
| **Activity period (*nocturnal)*** | -0.059 (-0.059, -0.058) | 0.026 (0.026, 0.026) | -2.279 (-2.296, -2.261) | 0.024 (0.023, 0.025) |
| Average ambient temperature | -0.002 (-0.002, -0.002) | 0.003 (0.003, 0.003) | -0.683 (-0.694, -0.672) | 0.496 (0.489, 0.503) |
| Seasonality ambient temperature | -0.006 (-0.006, -0.006) | 0.006 (0.006, 0.006) | -1.075 (-1.089, -1.061) | 0.284 (0.278, 0.291) |
| λ = 0.759 (0.756, 0.763); R^2^ = 0.770 (0.769, 0.770) | | | | |

Table S4. PGLS analyses of the effects of the ambient environment (including average ambient temperature, average NDVI and CV of NDVI) on brain size, while controlling for body temperature and other confounding effects. Variables with significant effects (P<0.05) are highlighted in in bold. The 95% confidence intervals for the 100 trees are shown in brackets.

|  | **Estimate** | **se** | **t** | **P** |
| --- | --- | --- | --- | --- |
| ***CV NDVI n = 148*** | | | | |
| Intercept | 1.011 (1.009, 1.012) | 0.205 (0.202, 0.209) | 4.946 (4.873, 5.018) | <0.001 (<0.001, <0.001) |
| **Body mass (log-10)** | 0.552 (0.552, 0.552) | 0.022 (0.022, 0.022) | 25.578 (25.529, 25.626) | <0.001 (<0.001, <0.001) |
| Taxon (Reptile) | 0.188 (0.186, 0.189) | 0.26 (0.254, 0.267) | 0.729 (0.714, 0.743) | 0.469 (0.46, 0.477) |
| **Activity period (nocturnality)** | -0.102 (-0.102, -0.101) | 0.043 (0.043, 0.043) | -2.361 (-2.372, -2.351) | 0.02 (0.019, 0.02) |
| Body temperature | 0.003 (0.003, 0.003) | 0.004 (0.004, 0.004) | 0.795 (0.772, 0.818) | 0.431 (0.417, 0.444) |
| Average ambient temperature | -0.002 (-0.002, -0.001) | 0.004 (0.004, 0.004) | -0.386 (-0.402, -0.371) | 0.701 (0.689, 0.712) |
| Average NDVI per year | 0.015 (0.014, 0.016) | 0.105 (0.104, 0.105) | 0.147 (0.135, 0.158) | 0.884 (0.875, 0.893) |
| CV NDVI per year | -0.003 (-0.003, -0.003) | 0.002 (0.002, 0.002) | -1.271 (-1.287, -1.256) | 0.207 (0.201, 0.213) |
| λ = 0.826 (0.811, 0.841); R^2^ = 0.836 (0.835, 0.836) | | | | |

Table S5. PGLS analyses of the effects of the ambient environment (including average and seasonality in ambient temperature) on brain size, while controlling for body temperature and other confounding effects. Variables with significant effects (P<0.05) are highlighted in in bold. The 95% confidence intervals for the 100 trees are shown in brackets.

|  | **Estimate** | **se** | **t** | **P** |
| --- | --- | --- | --- | --- |
| ***Ambient temperature n = 152*** | | | | |
| Intercept | 1.057 (1.055, 1.058) | 0.190 (0.187, 0.194) | 5.595 (5.496, 5.694) | <0.001 (<0.001, <0.001) |
| **Body mass (log-10)** | 0.552 (0.552, 0.553) | 0.021 (0.021, 0.021) | 26.341 (26.279, 26.403) | <0.001 (<0.001, <0.001) |
| Taxon (R*eptile*) | 0.185 (0.184, 0.186) | 0.252 (0.246, 0.258) | 0.744 (0.728, 0.761) | 0.459 (0.449, 0.469) |
| **Activity period (*nocturnal)*** | -0.107 (-0.107, -0.106) | 0.042 (0.042, 0.042) | -2.557 (-2.568, -2.546) | 0.012 (0.011, 0.012) |
| Body temperature | 0.004 (0.004, 0.004) | 0.004 (0.004, 0.004) | 1.025 (1.003, 1.046) | 0.310 (0.300, 0.320) |
| Average ambient temperature | -0.003 (-0.003, -0.003) | 0.004 (0.004, 0.004) | -0.683 (-0.695, -0.670) | 0.497 (0.489, 0.505) |
| Seasonality ambient temperature | -0.014 (-0.014, -0.014) | 0.009 (0.008, 0.009) | -1.632 (-1.644, -1.620) | 0.106 (0.103, 0.108) |
| λ = 0.817 (0.801, 0.833); R^2^ = 0.838 (0.838, 0.839) | | | | |

**Figures**

Figure S1. Mean ambient temperatures of nocturnal versus diurnal + cathemeral amphibians and reptiles in our sample

Figure S2. Mean body temperature of nocturnal versus diurnal + cathemeral amphibians and reptiles in our sample.

**Supplementary analyses**

Here we report on various additional analyses not reported in the main text because they did not affect our conclusions, but are included here to document this.

*Geographic range centroid*

In the main text, the analysis used the ambient temperature and NDVI calculated for the entire geographic range of each species. However, a species’ density tends to be higher around the centroid of the geographic range. Hence, we also calculated the average ambient temperature and CV and average NDVI from the species’ latitudinal centroids, which we adjusted by taking the values in an area of 2° latitude and longitude around this point, to remove any local biases. This different way of estimating climate or productivity did not change the result reported in the main text (Table SS1).

Table SS1. PGLS analyses of the effects of the ambient environment, calculated from latitudinal centroids, including average ambient temperature and average and CV of NDVI on brain size, while controlling for various confounding effects. Significant effects (P<0.05) in bold. The 95% confidence intervals for the 100 trees are shown in brackets.

|  | **Estimate** | **se** | **t** | **P** |
| --- | --- | --- | --- | --- |
| ***CV NDVI n = 349*** | | | | |
| Intercept | 0.972 (0.971, 0.973) | 0.164 (0.162, 0.165) | 5.940 (5.890, 5.990) | <0.001 (<0.001, <0.001) |
| **Body mass (log-10)** | 0.536 (0.536, 0.537) | 0.016 (0.016, 0.016) | 33.301 (33.276, 33.325) | <0.001 (<0.001, <0.001) |
| Taxon (Reptile) | 0.329 (0.328, 0.330) | 0.236 (0.234, 0.237) | 1.398 (1.387, 1.408) | 0.164 (0.161, 0.167) |
| **Activity period (nocturnality)** | -0.054 (-0.054, -0.054) | 0.026 (0.026, 0.026) | -2.070 (-2.086, -2.054) | 0.040 (0.038, 0.041) |
| Average ambient temperature (centroid) | -0.003 (-0.003, -0.003) | 0.002 (0.002, 0.002) | -1.096 (-1.111, -1.081) | 0.275 (0.269, 0.282) |
| Average NDVI per year (centroid) | 0.040 (0.040, 0.041) | 0.061 (0.061, 0.061) | 0.665 (0.656, 0.673) | 0.507 (0.501, 0.513) |
| CV NDVI per year (centroid) | -0.001 (-0.001, -0.001) | 0.001 (0.001, 0.001) | -0.979 (-0.988, -0.971) | 0.329 (0.324, 0.333) |
| λ = 0.746 (0.742, 0.750); R^2^ = 0.769 (0.769, 0.769) | | | | |

*Other aspects of the niche*

We also examined the effects of other aspects of the species’ ecological niche, such as foraging mode (sit-and-wait vs active foraging) and substrate (arboreal vs terrestrial or aquatic), because they all potentially affect the energy balance and thus may independently affect brain size. The results (TableSS2) show that substrate and foraging modes only have modest statistical effects on brain size at best, whereas the correlation with activity period remains strong.

Table SS2. PGLS analyses of the effects of the ambient environment (average ambient temperature, average and CV of NDVI) and aspects of the ecological niche (foraging mode and substrate) on brain size, while controlling for various confounding effects. Significant effects (P<0.05) in bold. The 95% confidence intervals for the 100 trees are shown in brackets.

|  | **Estimate** | **se** | **t** | **P** |
| --- | --- | --- | --- | --- |
| ***CV NDVI n = 231*** | | | | |
| Intercept | 0.993 (0.991, 0.994) | 0.171 (0.170, 0.173) | 5.808 (5.761, 5.855) | <0.001 (<0.001, <0.001) |
| **Body mass (log-10)** | 0.554 (0.554, 0.554) | 0.015 (0.015, 0.016) | 35.774 (35.716, 35.831) | <0.001 (<0.001, <0.001) |
| Taxon (Reptile) | 0.244 (0.243, 0.245) | 0.231 (0.229, 0.232) | 1.058 (1.050, 1.066) | 0.292 (0.288, 0.295) |
| **Activity period (nocturnality)** | -0.068 (-0.068, -0.067) | 0.028 (0.028, 0.028) | -2.393 (-2.409, -2.376) | 0.018 (0.017, 0.019) |
| Average ambient temperature (centroid) | 1e-04 (7e-05, 1e-04) | 0.002 (0.002, 0.002) | 0.044 (0.031, 0.056) | 0.950 (0.943, 0.957) |
| Average NDVI per year (centroid) | 0.028 (0.028, 0.029) | 0.059 (0.059, 0.059) | 0.482 (0.468, 0.497) | 0.631 (0.620, 0.641) |
| CV NDVI per year (centroid) | -0.001 (-0.001, -0.001) | 0.001 (0.001, 0.001) | -0.927 (-0.945, -0.910) | 0.357 (0.348, 0.366) |
| Foraging mode (sit-and-wait) | 0.040 (0.039, 0.040) | 0.026 (0.025, 0.026) | 1.548 (1.538, 1.558) | 0.124 (0.121, 0.126) |
| Substrate (terrestrial & aquatic) | -0.039 (-0.039, -0.038) | 0.024 (0.024, 0.025) | -1.583 (-1.595, -1.571) | 0.115 (0.113, 0.118) |
| λ = 0.880 (0.879, 0.882); R^2^ = 0.864 (0.863, 0.864) | | | | |

*Body plan effects*

Since the body plan (Bauplan), in the form of leg development (having or lacking legs), has a strong effect on relative brain size [1], it would make sense to control for the number of limbs. Leg development was dichotomized as ‘four-legged’ and ‘limbless’. One species, *Lerista bipes*, was reported as having hindlimbs only, and two other species as having reduced legs in Meiri [2]. We categorized them as limbless in our study.

We added body plan in the analysis of predictions 1 and 2. However, since the body plan is only variable within reptiles, specifically only in Sauria and Amphisbaenia, we had to replace the variable ‘Taxon’ by ‘body plan’ to avoid overfitting.

The results (Table SS3) are entirely consistent with those of Table 1 and Table S1, i.e., there was a significant effect of the interaction between activity period and mean body temperature, and in species having the opportunity to bask, relative brain size increased with Tb, whereas in nocturnal species it decreased (Table SS3).

Table SS3. PGLS analyses of the effects of mean Tb on brain size for diurnal & cathemeral (basking) and nocturnal (non-basking) species, respectively, while controlling for various confounding effects. Significant effects (P<0.05) in bold. The 95% confidence intervals for the 100 trees are shown in brackets.

|  | **Estimate** | **se** | **t** | **P** |
| --- | --- | --- | --- | --- |
| ***All species (n = 153)*** | | | | |
| *Intercept* | 0.957 (0.955, 0.959) | 0.125 (0.124, 0.126) | 7.671 (7.628, 7.713) | <0.001 (<0.001, <0.001) |
| **Body mass (log-10)** | 0.561 (0.561, 0.562) | 0.018 (0.018, 0.018) | 30.431 (30.387, 30.475) | <0.001 (<0.001, <0.001) |
| **Body plan (*Limbless*)** | -0.279 (-0.280, -0.278) | 0.064 (0.064, 0.064) | -4.342 (-4.365, -4.318) | <0.001 (<0.001, <0.001) |
| Activity period (AP, *nocturnal*) | 0.256 (0.255, 0.258) | 0.131 (0.131, 0.132) | 1.951 (1.939, 1.962) | 0.053 (0.052, 0.055) |
| Body temperature (Tb) | 0.005 (0.004, 0.005) | 0.004 (0.004, 0.004) | 1.224 (1.211, 1.237) | 0.224 (0.219, 0.229) |
| **AP (nocturnal)*Tb** | -0.015 (-0.015, -0.015) | 0.005 (0.005, 0.005) | -2.701 (-2.711, -2.692) | 0.008 (0.008, 0.008) |
| λ = 0.634 (0.624, 0.645); R^2^ = 0.867 (0.866, 0.867) | | | | |
| ***Basking species (n = 104)*** | | | | |
| *Intercept* | 0.970 (0.969, 0.971) | 0.105 (0.104, 0.105) | 9.252 (9.228, 9.276) | <0.001 (<0.001, <0.001) |
| **Body mass (log-10)** | 0.539 (0.539, 0.539) | 0.019 (0.019, 0.019) | 28.105 (28.077, 28.133) | <0.001 (<0.001, <0.001) |
| **Body plan (*Limbless*)** | -0.330 (-0.331, -0.329) | 0.069 (0.069, 0.069) | -4.766 (-4.777, -4.756) | <0.001 (<0.001, <0.001) |
| **Body temperature (Tb)** | 0.007 (0.007, 0.007) | 0.003 (0.003, 0.003) | 2.078 (2.066, 2.090) | 0.041 (0.039, 0.042) |
| λ = 0.505 (0.499, 0.510); R^2^ = 0.889 (0.889, 0.889) | | | | |
| ***Non-basking species (n = 49)*** | | | | |
| *Intercept* | 1.174 (1.172, 1.175) | 0.175 (0.173, 0.176) | 6.731 (6.688, 6.775) | <0.001 (<0.001, <0.001) |
| **Body mass (log-10)** | 0.621 (0.620, 0.621) | 0.044 (0.044, 0.044) | 14.147 (14.123, 14.170) | <0.001 (<0.001, <0.001) |
| **Body plan (*Limbless*)** | -0.199 (-0.200, -0.197) | 0.116 (0.115, 0.117) | -1.714 (-1.723, -1.705) | 0.094 (0.092, 0.095) |
| **Body temperature (Tb)** | -0.013 (-0.013, -0.013) | 0.006 (0.006, 0.006) | -2.122 (-2.136, -2.108) | 0.040 (0.039, 0.041) |
| λ = 0.703 (0.686, 0.719); R^2^ = 0.819 (0.819, 0.820) | | | | |

**References:**

1. de Meester G, Huyghe K, van Damme R. Brain size, ecology and sociality: a reptilian perspective. Biological journal of the linnean society. 2019;126:381–91.

2. Meiri S. Traits of lizards of the world: variation around a successful evolutionary design. Global Ecology and Biogeography. 2018;27:1168–72.
